# Growth Performance, Dietary Energetics, Blood Metabolites, Carcass Traits, Meat Quality, and Gene Expression of Lambs Supplemented with a Polyherbal Phytogenic Additive

**DOI:** 10.3390/vetsci11110520

**Published:** 2024-10-25

**Authors:** José Felipe Orzuna-Orzuna, Alejandro Lara-Bueno, Adrián Gloria-Trujillo, Germán David Mendoza-Martínez, Luis Alberto Miranda-Romero, Pedro Abel Hernández-García

**Affiliations:** 1 Departamento de Zootecnia, Universidad Autónoma Chapingo, Chapingo CP 56230, Mexico; jforzuna@gmail.com (J.F.O.-O.); microbiologia.pecuaria08@gmail.com (L.A.M.-R.); 2 Departamento de Producción Agrícola y Animal, Universidad Autónoma Metropolitana-Xochimilco, Mexico City CP 04960, Mexico; agloria@correo.xoc.uam.mx (A.G.-T.); gmendoza@correo.xoc.uam.mx (G.D.M.-M.); 3 Centro Universitario Amecameca, Universidad Autónoma del Estado de México, Amecameca CP 56900, Mexico; pedro_abel@yahoo.com

**Keywords:** finishing lambs, bioactive compounds, phosphatidylcholine, blood biochemistry, hematological profile, nutrigenomics

## Abstract

This study aimed to evaluate the effects of supplementation with a polyherbal phytogenic additive (PPA) on the productive performance, dietary energetics, blood metabolites, carcass traits, meat quality, and gene expression of finishing lambs. Thirty-six male Pelibuey lambs (23.61 ± 0.57 kg body weight (BW)) were housed in individual pens and assigned to four treatments (*n* = 9) with different doses of PPA: 0 (CON), 2.5 (PPAL), 5 (PPAM), and 7.5 (PPAH) g of PPA/kg of DM for 56 days. Average daily gain, dry matter intake, and observed dietary net energy for maintenance and weight gain increased linearly (*p* < 0.05) in lambs supplemented with PPAH. A linear reduction (*p* = 0.02) in FCR was detected in lambs fed PPAM and PPAH. The PPAH supplementation linearly increased (*p* < 0.001) *Longissimus dorsi* muscle area, but other carcass traits were not affected (*p* > 0.05) by PPA doses. The physicochemical characteristics of the meat and the hematological parameters of the lambs were not affected (*p* > 0.05) by the PPA doses. The glucose, uric acid, creatinine, and bilirubin serum concentrations decreased linearly (*p* < 0.05) in lambs supplemented with PPAM and PPAH. Gene ontology analyses showed that nine biological processes were modified (*p* < 0.05), including DNA replication, drug metabolism–cytochrome P450, oxidative phosphorylation, and chemical carcinogenesis–reactive oxygen species. In conclusion, high doses (7.5 g/kg DM) of PPA can improve growth performance and dietary energy utilization efficiency in finishing lambs. Likewise, gene expression analysis indicates that supplementation with high doses of PPA could improve energy production and antioxidant status in finishing lambs.

## Simple Summary

The inclusion of phytogenic additives in diets for ruminants has shown a positive impact on the growth and health of animals. However, information on the effects of phytogenic additives on dietary energetics, meat quality, and gene expression in ruminants is limited. This study aimed to evaluate the effects of a polyherbal phytogenic additive on the growth performance, dietary energetics, carcass and meat traits, blood metabolites, and gene expression of finishing lambs. The polyherbal phytogenic additive improves feed intake, weight gain, feed efficiency, and dietary energy utilization efficiency. Likewise, the effects observed at the genetic level indicate that the polyherbal phytogenic additive improves energy production and antioxidant status in finishing lambs.

## 1. Introduction

Since the 1950s, antibiotics have been used successfully in preventing, controlling, and treating infectious diseases in domestic animals [1]. Furthermore, the inclusion of low doses of antibiotics in livestock feeds has been documented to improve the health, feed efficiency, growth rate, and carcass quality of animals [2,3]. However, the irrational and irresponsible use of antibiotics has contributed to the emergence of bacterial strains resistant to their effects [1]. This effect has led to the prohibition of antibiotics as growth promoters in several countries and has increased interest in the search for natural products that improve animal health and performance [4]. Among the natural products currently available are phytogenic additives, which, according to Windisch et al. [5], are products derived from plants that can be used in livestock feeding to improve their productivity.

Among the phytogenic additives are polyherbal phytogenic additives (PPAs), which consist of plant mixtures and differ from plant extracts in that they contain several bioactive metabolites with pharmaceutical properties [6]. Previous studies have shown the positive impact of dietary supplementation with PPAs on animal performance [7], dietary energy utilization efficiency [8], and meat quality [9] of finishing lambs. Other PPAs have been successfully used to improve the production of volatile fatty acids in the rumen [10], intestinal morphology and immune response [11], and the antioxidant status of blood serum in growing lambs [12]. BioCholine^®^ is a PPA made from medicinal plant parts of *Andrographis paniculata*, *Azadirachta indica*, *Achyrantes aspera*, and *Trachyspermum ammi*, which is standardized to contain 16 g of phosphatidylcholine (PtdCho) per kilogram of product.

In non-ruminants, individual supplementation with *A. aspera* or *T. ammi* has been reported to improve animal performance, feed digestibility, and intestinal microbiota [13,14]. However, based on the literature reviewed, no previously published information exists on the effects of *A. aspera or T. ammi* in ruminants. Dietary inclusion of *A. paniculata* has been successfully used to improve rumen parameters, rumen microbiota, and meat quality in goats [15,16]. Previous studies [17,18] show the positive impact of supplementation with A. indica on nutrient digestibility, weight gain, and carcass traits of small ruminants. According to Hassan et al. [19], plant combinations provide complex mixtures of bioactive compounds with synergistic effects. Therefore, using a PPA containing a combination of *A. aspera*, *T. ammi*, *A. paniculata*, and A. indica could be a better strategy to improve livestock health and productivity than the individual administration of those plants.

On the other hand, it has been documented that dietary supplementation with products containing PtdCho improves animal health and performance in non-ruminants through changes in gene expression [20,21]. Likewise, Diaz et al. [22] reported that a PPA containing PtdCho improved calves’ growth rate and health status through changes in the expression of lipid, carbohydrate, and protein metabolism genes. Other products containing PtdCho have shown a positive impact on rumen microbial populations [23], the antioxidant status of blood serum [24], and the nutrient digestibility [25] of ruminants. However, based on the literature reviewed, no information is available on the effects of dietary supplementation with PPAs or PtdCho on the gene expression of finishing lambs.

Based on the above background, we hypothesize that supplementation with a PPA made from medicinal plants and containing PtdCho will benefit the animal performance, blood metabolites, meat and carcass quality, and gene expression of finishing lambs. The objective of this study was to evaluate the effects of increasing levels of a polyherbal phytogenic additive made from medicinal plants (*Andrographis paniculata*, *Azadirachta indica*, *Achyrantes aspera*, and *Trachyspermum ammi*) and containing phosphatidylcholine on the productive performance, dietary energetics, blood metabolites, carcass traits, meat quality, and gene expression of finishing lambs.

## 2. Materials and Methods

### 2.1. Experimental Location

The present experiment was carried out from June to August 2022 at the Experimental Farm of the Universidad Autónoma Chapingo, located in Texcoco, State of Mexico, Mexico (Latitude: 19°30′45″ N; Longitude: 98°52′47″ W; altitude: 2250 masl). The average annual temperature of Texcoco is 18.2 °C, and the average annual precipitation is 665 mm [8]. All lamb care procedures were carried out following the federal guidelines for the use and care of animals [26] and were approved by the Research Ethics and Bioethics Committee of the Autonomous University of the State of Mexico (Protocol #25-05 2022).

### 2.2. Characterization of the Polyherbal Phytogenic Additive

The polyherbal phytogenic additive (PPA) used was BioCholine^®^ (Nuproxa S. de RL. de CV., Querétaro, México). BioCholine^®^ is a commercial product composed of plant parts of *Andrographis paniculata*, *Azadirachta indica*, *Achyrantes aspera*, and *Trachyspermum* ammi, and is standardized to contain 16 g of phosphatidylcholine (PtdCho) per kilogram of product. The methodology previously described by other authors [6,27] was followed to extract and characterize the bioactive compounds of the PPA used. Briefly, bioactive compounds were extracted from PPA using an ultrasonic processor (GEX130, 115 V 50/60 Hz) equipped with a 3 mm titanium tip and mechanical stirrers (Cole-Parmer, Vernon Hills, IL, USA). Subsequently, one gram of PPA was mixed with 10 mL of hexane, and the organic phase was separated, concentrated to 1 mL, and then evaporated with a rotary evaporator (model R-200, Büchi, Flawil, Switzerland) to obtain the necessary material for the final analysis.

The characterization of the PPA was carried out by gas chromatography (Agilent 7890B, Agilent, Santa Clara, CA, USA) coupled to mass spectrophotometry (Agilent 5977A), equipped with a capillary column of a 30 m length, 250 μm diameter, and 0.25 μm film thickness (HP-5MS Ultra Inert, Agilent, Santa Clara, CA, USA). The temperature program was 40 °C for 3 min, and then increased to 250 °C at a rate of 10 °C/min and held there for 36 min. The temperature used in the injector was 240 °C in split mode, the helium flow rate was 1 mL/min, and the mass spectrophotometry was programmed in SCAN mode (35–550 *m*/*z*) to identify the PPA compounds. Table 1 shows the main compounds detected in PPA (BioCholine^®^).

### 2.3. Animals, Experimental Design, and Diet Composition

Thirty-six male Pelibuey lambs (23.61 ± 0.57 kg BW, 4–5 months old) were distributed using a completely randomized experimental design into one of four treatments (*n* = 9): (1) basal diet without PPA (CON); (2) PPAL, CON + 2.5 g of PPA/kg of dry matter (DM); (3) PPAM, CON + 5 g of PPA/kg DM; and (4) PPAH, CON + 7.5 g of PPA/kg DM. The 5 g dose of PPA (BioCholine^®^) was chosen based on a previous experiment in which this dose improved rumen propionate concentration and antioxidant status in the blood serum of growing lambs [24]. However, the animal performance of dairy cows and calves supplemented with the same PPA used in the present study has shown dose-dependent responses [22,25]. Therefore, the PPA doses in the present study included a low dose (2.5 g/kg DM) and a high dose (7.5 g/kg DM). All lambs were adapted to individual pens and the basal diet for 12 days and were subsequently subjected to an experimental phase of 56 days. At the beginning of the adaptation period, all lambs received a subcutaneous administration of 0.5 mL/lamb of ivermectin (Closantil^®^ 5%, Chinoin Labs, Mexico City, Mexico), an intramuscular injection with 500,000 IU of vitamin A, 75,000 IU of vitamin D, and 50 mg of vitamin E (Vigantol^®^, Bayer Labs, Mexico City, Mexico), and were vaccinated intramuscularly against Pasteurella and Clostridium (2.5 mL/lamb, Multibacterina^®^ 7, MSD Labs, Mexico City, Mexico). The individual pens had an area of 2 m^2^ with walls (1.2 m high) and a concrete floor, galvanized sheet shade (2 m high), automatic waterer, and individual feeder (40 cm bunk space/lamb).

**Figure 1:**
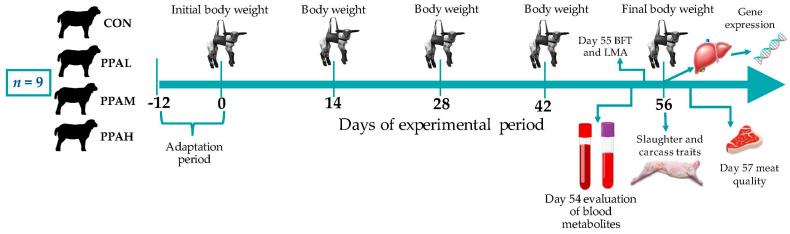
Diagram of the experimental design and the samplings carried out during the experiment. CON: basal diet without polyherbal phytogenic additive (PPA); PPAL, CON + 2.5 g of PPA/kg dry matter (DM); PPAM, CON + 5 g of PPA/kg DM; and PPAH, CON + 7.5 g of PPA/kg DM. BFT: back fat thickness; LMA: *Longissimus dorsi* muscle area.

PPA was supplied to the lambs through the basal diet, formulated to obtain weight gains of 300 g/d [28]. The ingredients used and the nutritional composition of the experimental diets are shown in Table A1. First, PPA doses (2.5, 5, or 7.5 g/kg DM) were mixed with the minor ingredients of the diet (mineral premix, calcium carbonate, and common salt). Subsequently, these amounts were mixed with the rest of the ingredients until the complete experimental diets were obtained. Food was offered every day at 7:00 a.m. and 3:00 p.m. To guarantee ad libitum consumption, an amount of food 5% greater than the previous day’s consumption was always assigned. Water was available ad libitum throughout the experiment. Figure 1 shows the experimental design used and the sampling carried out.

Samples of the experimental diets were collected weekly and stored in polyethylene bags at −20 °C. Before chemical analyses, subsamples were thawed for 12 h at room temperature. Subsequently, equal amounts of the subsamples collected weekly were mixed to obtain a composite sample. Samples were dried at 55 °C for 72 h in a forced-air oven and then ground with a Wiley mill (model 4, Arthur Thomas Co., Philadelphia, PA, USA) using a 1 mm sieve. Finally, the contents of dry matter, crude protein, ether extract, and ash were determined following the procedures described by AOAC [29]. The contents of neutral detergent fiber and acid detergent fiber were determined using the methods of Van Soest et al. [30].

### 2.4. Calculations

In the experimental phase, the body weight (BW) of each of the lambs was recorded one hour before morning feeding on days 1, 14, 28, 42, and 56 using a WeiHeng^®^ brand digital scale (model WH-C100, Guangzhou Weiheng Electronics Co., Ltd., Guangzhou, China). The BWs recorded on days 1, 14, 28, and 42 were converted to reduced body weight (SBW) with the equation [31] SBW = BW × 0.96 to adjust for gastrointestinal filling. Before recording the final body weight (FBW) on day 56, all lambs were fasted for 18 h. Consequently, the dietary net energy and weight gain estimates were made based on SBW. Daily weight gain (ADG, kg/d) was calculated for periods of 14 days using the SBW data recorded on days 1, 14, 28, 42, and 56 with the following equation: ADG = (final SBW − initial SBW)/14. Each lamb’s feed offered and rejected was recorded daily to estimate dry matter intake (DMI, kg/d). The lambs’ feed conversion ratio (FCR) was calculated with the following equation: FCR = DMI/ADG.

Previous studies [8,32] mention that, in animal performance trials with lambs, the relationship between observed and expected DMI and the relationship between observed and expected dietary net energy (NE) serve as indicators to evaluate the efficiency of dietary energy utilization. The expected DMI estimate was based on the average ADG and SBW observed and the NE values calculated for the experimental diets (Table A1). For this, the following equation was used: expected DMI, kg/d = (EM/NEm) + (EG/NEg), where EM (energy required for maintenance, expressed in Mcal/d) = 0.056 × SBW^0.75^ [NRC, 1985] and EG (energy gain, Mcal/d) = ADG × 0.276 × SBW^0.75^ [33]. The values of NEm (NE maintenance, Mcal/kg of DM) and NEg (NE gain, Mcal/kg of DM) were 1.81 and 1.26, respectively, and were calculated with tabular values based on the ingredient composition of the experimental diets [28]. The coefficient of 0.276 was taken from the NRC [33], assuming a medium mature weight for male Pelibuey lambs [34]. Finally, with the DMI values observed in the present study and the ME and EG values, the observed dietary NE was calculated using the quadratic formula x = (−b − √(b^2 − 4ac))/2c, where x = NEm (Mcal/kg), a = −0.41 ME, b = 0.877 ME + 0.41 DMI + EG, and c = −0.877 DMI [35].

### 2.5. Carcass Traits, Carcass Morphometry, and Non-Carcass Components

Back fat thickness (BFT) and Longissimus dorsi muscle area (LDMA) were measured on day 55 of the experimental phase with the procedures described by Silva et al. [36]. For this, all the lambs were shaved between the 12th and 13th ribs, and subsequently a Sonovet 600 brand ultrasound (Medison, Inc., Cypress, CA, USA) with a 7.5 Mhz transducer was placed. The yield grade (YG) of the carcass was estimated with the equation YG = 0.4 + (10 × BFT, in cm) [37].

After obtaining FBW on day 56 of the experiment, all lambs were slaughtered on the same day according to the procedures of the Mexican Official Standard [38]. Immediately after completing the slaughter and bleeding of the lambs, the head, legs, skin, and all internal organs were separated from the carcass, and the hot carcass weight (HCY) was recorded. The equation HCY = (HCW/FBW) × 100 was used to estimate the hot carcass yield (HCY). Subsequently, all carcasses were cooled for 24 h at 4 °C, weighed individually, and then the cold carcass weight (CCW) was recorded. The cold carcass yield was estimated with the CCY = (CCW / FBW) × 100 equation.

For each of the lambs, the individual weight of the legs, skin, head, empty stomach complex (rumen, reticulum, omasum, and abomasum), empty small intestine (duodenum, jejunum, and ileum), empty large intestine (cecum, colon, and rectum), liver, kidneys, lungs, heart, and spleen were recorded. With the procedures described by Yañez et al. [39], in the cold carcass of each lamb, the following morphometric measurements were taken: internal carcass length (ICL), external carcass length (ECL), leg perimeter (LP), leg length (LL), chest girth (CG), and carcass compactness index (CCI), where CCI (kg/cm) = CCW/ILC.

### 2.6. Meat Quality

After keeping the carcasses at 4 °C for 24 h, the right *Longissimus dorsi* muscle (approximately 600 g) between the 4th and 12th rib was removed from each lamb with a scalpel. Subsequently, all samples were stored in identified polyethylene bags and frozen at −20 °C for one week. Before meat quality analyses, all samples were thawed at 4 °C for 24 h, and a scalpel was used to remove fat and subcutaneous tissue from each sample. Cooking loss (CL) was determined in duplicate following the procedures previously described by other authors [9,40]. Briefly, two 2.5-cm thick fillets were cut from each sample and grilled with an electric grill (Toastmaster cool-edge grill, Macon, MO, USA). The internal temperature of the fillets was monitored using a Taylor^®^ brand thermometer (model 99878, Seattle, WA, USA), which was placed at the geometric center of each sample. When the internal temperature reached 70 °C, the samples were removed from the rack and cooled to room temperature (19–24 °C). Finally, CL was calculated with the equation [40] CL (%) = ((Wr − Wc)/Wr) × 100, in which Wr is the raw weight and Wc is the cooked weight of the meat samples used.

Meat color was evaluated with the procedures described by Miltenburg [41]. Approximately 3 cm slices were first cut from each sample, and the freshly cut surfaces were exposed to atmospheric air in a laboratory room for 30 min at room temperature. Subsequently, lightness (L*), redness (a*), and yellowness (b*) were measured in triplicate in different positions throughout the sample with a HunterLab brand colorimeter (Model MiniScan XE Plus, Reston, VA, USA).

To determine the chemical composition of the meat, a 300 g sample was taken from each lamb and ground individually with a Ship to Shore brand meat grinder (Model 99598, Camarillo, CA, USA) until a homogeneous mixture was obtained [40]. After this, the content (g/100 g) of moisture, fat, protein, and collagen was determined in triplicate with a FOSS brand near-infrared spectrophotometer (FoodScan™ Lab, Hillerod, Denmark), according to the procedures described by Anderson [42].

### 2.7. Blood Metabolites

In the last week of the experimental phase (day 54), two blood samples were taken from the jugular vein of each lamb before morning feeding (06:30 h). The first blood sample (4 mL/lamb) was collected using BD Vacutainer^®^ K2 EDTA anticoagulant tubes (Becton, Dickinson and Company, Franklin Lakes, NJ, USA). These samples were transported at 4 °C for approximately 1 h to the laboratory, where they were analyzed with an EasyVet^®^ hematology analyzer (QS Kontrolab, Hamburg, Germany), as reported by Dorantes-Iturbide et al. [8]. The hematological variables obtained included hematocrit, complete blood count, and differential leukocyte count.

The second blood sample (6 mL/lamb) was collected with BD Vacutainer^®^ anticoagulant-free tubes (Becton, Dickinson and Company, Franklin Lakes, NJ, USA). These samples were centrifuged with a refrigerated centrifuge (Sigma 2–16 k, Sigma Laborzentrifugen GmbH, Osterode am Harz, Germany) at 3500 rpm for 20 min to obtain blood serum, which was stored at −20 °C in Eppendorf tubes. Finally, an EasyVet^®^ autoanalyzer (QS Kontrolab, Hamburg, Germany) was used to determine the contents of glucose, cholesterol, triglycerides, total protein, albumin, globulin, urea, uric acid, creatinine, bilirubin, liver enzymes (alkaline phosphatase, lactate dehydrogenase, and aspartate aminotransferase), calcium, and phosphorus in the blood serum, as reported in previous studies [6,7,40].

### 2.8. Liver Samples, RNA Extraction and Microarrays

Immediately after being slaughtered, liver samples were taken from the CON- and PPAH-supplemented lambs to extract RNA. For this, samples of 100 mg of liver tissue were taken in triplicate and placed in 2 mL microtubes (cap with O-ring) with 1 mL of DNA/RNA Shield™ reagent (Zymo Research, Irvine, CA, USA). The samples were placed in a cooler for transport to the laboratory. Subsequently, RNA extraction was carried out with the phenol–chloroform method, following the step-by-step protocol described by Toni et al. [43]. The integrity and purity of the RNA were evaluated following the procedures described in a previous study by our research team [22].

From each experimental treatment (CON and PPAH), a group of 30,000 ng of RNA was obtained. The replicates (lambs) contributed an equal concentration to form the total RNA in each group. Transcriptome analysis was performed using a heterologous mouse chip (M22K) with 24,341 applications at the Microarray Unit facilities of the Institute of Cellular Physiology of the National Autonomous University of Mexico (UNAM). GenArise software was used to identify differentially expressed genes (DEGs; z score 1.5) on the microarray. Furthermore, the bioinformatics tool DAVID 6.8 (Database for Annotation, Visualization, and Integrated Discovery) was used to evaluate gene sets’ enrichment, as Sheman and Lempicki suggested [44]. Enrichment analysis of gene sets through gene ontology was used to summarize the biological meaning of the identified differentially expressed transcripts, as reported by recent studies [21,22].

### 2.9. Statistical Analysis

#### 2.9.1. Performance, Dietary Energetics, Blood Metabolites, Carcass Traits, Non-Carcass Components and Meat Quality

All statistical analyses were performed using SAS statistical software [45]. First, the Shapiro–Wilk normality test was applied to all variables evaluated with the PROC UNIVARIATE procedure. Data on productive performance and dietary energetics were analyzed with the PROC MIXED procedure, using a completely randomized experimental design with measures repeated over time, in which each lamb was considered an experimental unit. The initial BW covariate was not included in the statistical model because it was not significant (*p* > 0.05). The statistical model was adjusted by testing different variance–covariance structures, and the best fit was obtained with the compound symmetry structure because the lowest values of AIC and BIC were detected [46]. The structure of the statistical model used was

Y*_ijk_* = *µ* + T*_i_*+ P*_j_*+ (T × P)*_ij_* + L_k_ + e*_ijk_*

where Y*_ijk_* = value observed in treatment *i* and period *j* for lamb k; *µ* = overall mean; T*_i_* = fixed effect of the ith treatment; P*_j_* = fixed effect of the *j*th period (period 1: 1–14, …, period 4: 43–56 d); (T × P)*_ij_* = fixed effect of the interaction between the *j*th treatment and the *i*th period; L_k_ = random effect of the lamb (repetition) within the treatment (k = 1, 2, 3,..., 36); and e*_ijk_* = random error.

Data on carcass traits, organs, meat quality, and blood metabolites were analyzed with the PROC GLM procedure, using a completely randomized experimental design where each lamb was considered an experimental unit. The FBW covariate was not included in the statistical model because it was not significant (*p* > 0.05). The statistical model used was the following:

Y_ij_ = µ + T_i_ + e_ij_

where Y_ij_ represents the observations, µ = general mean, T_i_ = fixed effect of the ith treatment, and eij = random error.

In all the variables evaluated, polynomial orthogonal contrasts were used to determine the linear and quadratic effects of the inclusion levels (0, 2.5, 5, and 7.5 g/kg DM) of PPA [47]. Significant differences were declared when *p* ≤ 0.05.

#### 2.9.2. Gene Enrichment Analysis

The gene enrichment analysis was performed considering at least two genes for each biological group, as reported by Mendoza-Martínez et al. [21]. Subsequently, to identify the enriched annotation terms that belonged to the tested gene sets and the biological closeness between the enriched processes, an EASE (modified Fisher Exact) score with *p* ≤ 0.05 and the fold enrichment value were used [44], respectively.

## 3. Results

### 3.1. Growth Performance and Dietary Energetics

Compared to the CON treatment, ADG and DMI increased linearly (*p* < 0.05) in lambs fed PPAH (Table 2). In contrast, a linear reduction (*p* = 0.02) was detected in the FCR of lambs fed PPAM and PPAH compared to CON lambs. However, the variation in DMI was not affected by the doses of PPA added to the diets (*p* > 0.05).

Compared to the CON treatment, observed dietary NE for maintenance (ObsNEm) and weight gain (ObsNEg) increased linearly (*p* < 0.05) in lambs fed PPAH (Table 2). Similarly, the relationship between observed and expected dietary NE for maintenance (OExNEm) and weight gain (OExNEg) increased linearly (*p* = 0.02) in lambs fed PPAH compared to CON lambs. In contrast, a linear reduction (*p* = 0.01) in the ratio between observed and expected DMI was detected for lambs fed PPAH compared to CON lambs.

### 3.2. Carcass Traits and Carcass Morphometry

Table 3 shows that LDMA increased linearly (*p* < 0.001) compared to the CON treatment in lambs fed PPAM and PPAH. However, PPA supplementation did not affect BFT, HCW, HCY, CCW, CCY, YG, ELC, ILC, CG, LL, PL, and CI.

### 3.3. Non-Carcass Components and Meat Quality

The weights of the stomach complex, small intestine, large intestine, lungs and trachea, heart, liver, kidneys, spleen, testicles, skin, head, and feet were not affected (*p* > 0.05) by the doses of PPA added to the diets (Table 4).

Table 5 shows that being supplemented with increasing doses of PPA did not affect CL, L*, a*, b*, protein, fat, moisture, and collagen content of meat (*p* > 0.05).

### 3.4. Blood Metabolites

Dietary supplementation with increasing doses of PPA did not affect (*p* > 0.05) blood levels of hematocrit, hemoglobin, red blood cells, mean corpuscular volume, mean corpuscular hemoglobin, mean corpuscular hemoglobin concentration, platelets, leukocytes, lymphocytes, monocytes, segmented neutrophils, band neutrophils, basophils, and plasma protein (Table 6).

Compared with CON treatment, serum concentrations of glucose, uric acid, creatinine, and bilirubin decreased linearly (*p* < 0.05) in lambs fed PPAM and PPAH (Table 7). However, serum concentrations of cholesterol, triglycerides, urea, total protein, albumin, globulin, albumin–globulin ratio, alkaline phosphatase, lactate dehydrogenase, aspartate aminotransferase, calcium, and phosphorus were not affected by the doses of PPA added to diets.

### 3.5. Gene Expression

Compared to CON treatment, PPAH supplementation modified the expression of 2312 genes at least 1.5 times, of which 1135 and 1177 were downregulated and upregulated, respectively. Table 8 shows that the biological processes enriched with the downregulated DEGs were DNA replication (*p* = 0.003), tyrosine metabolism (*p* = 0.006), metabolism of drugs (*p* = 0.003), the intestinal immune network for immunoglobulin A production (*p* = 0.009), TGF-β signaling pathway (*p* = 0.007), and amoebiasis (*p* = 0.0056). On the other hand, Table 9 shows that DEGs upregulated the biological processes of non-alcoholic fatty liver disease (*p* = 0.00003), oxidative phosphorylation (*p* = 0.0033), and chemical carcinogenesis–ROS (*p* = 0.0016).

## 4. Discussion

### 4.1. Growth Performance and Dietary Energetics

In the present study, the higher DMI observed could be associated with the presence of nonanoic acid in the PPA used since, according to recent studies [48,49], this acid can be used as a flavoring in foods for domestic animals with positive effects on food intake. Likewise, PPAH supplementation decreased (fold change = −2.11) the expression of the *Tas2r119* gene (taste receptor, type 2, member 119). Lower expression of *Tas2r119* in mammals decreases cholecystokinin production in neuroendocrine cells and leads to higher food intake [50]. In contrast, PPA doses did not affect the variation in DMI between individuals, which indicates that the evaluated PPA does not alter the well-being of the lambs [8,51]. On the other hand, in the current study, higher DMI and ObsNEg were observed in response to PPAH supplementation. Furthermore, previous studies have reported that some bioactive metabolites (carvacrol and PtdCho) and plants (*A. paniculata*) present in the PPA evaluated in the current study increase nutrient digestibility in ruminants [8,15,25]. These effects could result in greater metabolic availability of nutrients and energy to form new body tissues, which would explain the better ADG and FCR detected in PPAH lambs.

In the current study, higher ObsNEm, ObsNEg, OExNEm, and OExNEg were observed in lambs fed the PPAH diet. Similarly, Dorantes-Iturbide et al. [8] observed between 17.7 and 23.2% higher ObsNEm, ObsNEg, OExNEm, and OExNEg in finishing lambs supplemented with low doses (1 and 2 g/kg DM for 56 days) of PPA based on *A. paniculata*, *A. indica*, *Tinospora cordifolia*, *Ocimum sanctum*, and *Whitania somnifera*. The increases in ObsNEm, ObsNEg, OExNEm, and OExNEg detected in lambs fed PPAH could be related to the positively regulated DEGs within the oxidative phosphorylation process since, according to Sieck et al. [52], increased enzymatic activity in oxidative phosphorylation increases energy (ATP) production in ruminant cells. Furthermore, the presence of carvacrol in the PPA used could explain the positive effects detected in ObsNEm, ObsNEg, OExNEm, and OExNEg in the present study. This is because, in finishing lambs, carvacrol increases the rumen production of total volatile fatty acids, which are the primary source of energy in small ruminants [53]. Furthermore, the values of OExNEm and OExNEg detected in lambs fed with PPAH were greater than 1.0, which indicates greater efficiency in the use of dietary energy for maintenance and weight gain [8,54].

### 4.2. Carcass Traits and Carcass Morphometry

In vivo measurements of LDMA and BFT, and some carcass traits, such as HCW, HCY, CCW, and CCY, are important in the sheep meat industry because they influence the yield of prime cuts (e.g., loin and anterior ribs) [55,56]. In the current study, LDMA increased in response to PPAM and PPAH supplementation; however, BFT, HCW, HCY, CCW, CCY, and YG were not affected by increasing levels (2.5, 5.0, and 7.5 g/kg DM) of PPA added to the diets. The increase in LDMA observed in lambs supplemented with PPAM and PPAH is positive since, according to a recent study [57], LDMA is positively correlated (r = 0.43) with total muscle content in the lamb carcass. The increase in LDMA could be associated with the greater expression of the *Lep* gene observed in the current study since, according to Rosales et al. [58], the *Lep* gene encodes leptin, and serum levels of this hormone have a positive correlation (r = 0.55) with muscle accumulation in lambs.

Morphometric measurements in the sheep carcass can be used in equations to predict the amount of adipose tissue, muscle, and bone [57,59]. In the current study, PPA doses did not affect ELC, ILC, CG, LL, PL, and CI. In recent studies, Dorantes-Iturbide et al. [8] and Orzuna-Orzuna et al. [7] also did not detect changes in ELC, ILC, CG, LL, and PL of finishing lambs supplemented with various doses (between 1 and 3 g/kg DM) of different PPAs in the diet.

Our study’s growth performance and carcass trait results imply that high doses (7.5 g/kg DM) of BioCholine^®^ PPA can successfully improve growth rate, feed efficiency, and rib-eye cut quantity in finishing lambs. These effects could lead to increased economic profitability of the fattening process and positively impact the economic income of sheep producers and the meat industry.

### 4.3. Non-Carcass Components and Meat Quality

In the present study, the weights of lambs’ internal and external organs were not affected by supplementation with increasing doses of PPA. These effects indicate that the PPA used can be consumed by finishing lambs without affecting the development of their organs and body tissues [60]. Several previous studies [7,8,9] also did not detect changes in the weights of internal and external organs of finishing lambs supplemented with increasing doses (between 1 and 3 g/kg DM) of different PPAs containing *A. paniculata*, *A. indica*, *Acacia concinna*, among others.

From the point of view of meat science, the pH, CL, color, and chemical composition of meat influence the quality of sheep meat [61]. In the present study, the doses of PPA evaluated did not alter the pH, CL, color (L*, a*, and b), or chemical composition of the meat, which suggests that the PPA used can be included in lamb diets without affecting the quality of the meat. Previous studies also did not detect changes in pH, CL, color, and chemical composition of meat from finishing lambs supplemented with a PPA (1, 2, and 3 g/kg DM for 56 days) based on *A. concinna*, *Saccharum officinarum*, and *Balanites roxburghi* [9] or with a PPA (5, 10, and 15 g/kg DM for 60 days) based on *Emblica officinalis* and *Ocimum sanctum* [51].

### 4.4. Blood Metabolites

According to Roland et al. [62], hematological parameters are useful to evaluate health in ruminants because they allow the detection of systemic diseases and disorders of the hematological system. In the current study, the lambs’ hematological parameters were unaffected by the PPA doses used. Likewise, the average values of all hematological parameters were within the range reported as normal for clinically healthy growing male Pelibuey lambs [63]. These results indicate that the evaluated PPA can be included in diets for finishing lambs without altering the hematological system of the animals. Similarly, previous studies reported that dietary inclusion of other PPAs (between 1 and 15 g/kg DM) with different combinations of plants that included *A. paniculata*, *A. indica*, *A. concinna*, *S. officinarum*, *B. roxburghi*, *E. officinalis*, and *O. sanctum* also did not affect the normal ranges of hematological parameters in lambs [8,64] and calves [6,27].

Braun et al. [65] indicate that, in sheep, blood chemistry parameters are mainly used as a diagnostic tool for nutritional, liver, and muscle disorders. In the current study, supplementation with PPAM and PPAH reduced glucose, uric acid, creatinine, and bilirubin serum concentrations. However, the average values of these and the other serum metabolites were within the range reported as normal for clinically healthy lambs [66]. This effect suggests that the evaluated PPA can be used in lamb diets without the risk of causing nutritional, liver, and muscle disorders. The lower serum glucose detected in the PPAM and PPAH lambs could be related to the presence of oleic acid in the PPA used since, according to a recent study [67], the intake of oleic acid decreases serum glucose in ruminants through an increase in serum insulin levels. The PPA evaluated also contains p-cymene, an aromatic monoterpene with hypoglycemic effects in mammals [68]. Furthermore, the lower serum concentration of uric acid observed in the current study could be explained by the presence of thymoquinone in the PPA used. This is because thymoquinone inhibits the activity of the enzyme xanthine oxidase, which is necessary to produce uric acid through the breakdown of purines [69].

On the other hand, the lower serum creatinine detected in the current study could be associated with the high linoleic acid content in the PPA evaluated. This is because linoleic acid consumption increases the glomerular filtration rate [70], which is inversely related to serum creatinine [71]. Furthermore, the PPA used in this study contains terpenes (carvacrol and p-cymene), which can inhibit the expression of the *UGT1A9* gene (member A9 of family 1 of UDP glucuronosyltransferase) [72]. This mechanism could explain the lower serum bilirubin observed in lambs fed with PPAM and PPAH since, according to Zhou et al. [73], the *UGT1A9* gene is necessary for the formation of direct and conjugated bilirubin through the glucuronidation process.

### 4.5. Gene Expression

In the current study, dietary PPAH supplementation decreased the expression of DNA damage-related genes within the biological process of DNA replication. Among these genes, *Prim2* encodes to produce a DNA replication-promoting protein, mismatch repair, and cell activation [74]. Likewise, the genes *Rfc1*, *Rfc3*, and *Rfc4* encode replication factors C that participate in the excision repair of damaged DNA [75]. The gene *Mcm4* encodes one of the six proteins that make up the mini-chromosome maintenance complex essential for DNA strand separation during chromosome replication, an essential process for cell viability [76]. On the other hand, the *Rnaseh2b* gene encodes an RNase H2 responsible for initiating the ribonucleotide excision repair pathway, which is incorporated into genomic DNA in large quantities during replication, causing DNA destabilization [77]. Finally, the *Pold1* gene encodes the catalytic and error-correcting subunit of DNA polymerase delta, which is responsible for the synthesis of lagging-strand DNA during DNA replication [78]. These effects of PPAH intake could improve DNA integrity in lambs, and good DNA integrity is positively associated with better health status in ruminants [22].

The second most down-enriched biological process was tyrosine metabolism, within which several genes (*Aldh3a1*, *Adh4*, *Aox1*, *Fahd1*, *Got2*, *Maoa*) with amino acid oxidative activity are grouped [79]. Among these genes, the *Maoa* gene encodes for the production of monoamine oxidases, which produce hydrogen peroxide (H_2_O_2_) and reactive oxygen species (ROS) when they metabolize dopamine in the cytosol of cells [80]. According to Ahmed and Younus [79], the *Aldh3a1* and *Adh4* genes encode to produce alcohol dehydrogenase and aldehyde dehydrogenase enzymes, which can reduce and inactivate H_2_O_2_ and ROS in cells. This effect suggests that dietary supplementation with PPAH could help to decrease oxidative stress (OS) in finishing lambs.

Within the biological process of drug metabolism cytochrome P450, dietary supplementation with PPAH decreased the expression of the genes *Gsta3*, *Gsta4*, *Gstm3*, *Fmo1*, and *Fmo2*. The genes *Gsta3*, *Gsta4*, and *Gstm3* belong to the detoxification enzyme family Glutathione S-Transferase, and a reduction in their expression could decrease the conjugation of glutathione into toxins for their excretion [81]. Likewise, the genes *Fmo1* and *Fmo2* encode for NADPH-dependent flavin monooxygenases, which are enzymes that catalyze the oxidation of drugs, pesticides, and xenobiotics [82].

Within the intestinal immune network for IgA production, dietary supplementation with PPAH decreased the expression of the genes *Tgfβ1*, *Cd40lg*, *Il6*, *Tnfsf13*, *H2-Oa*, *Cxcl12*, and *Tnfrsf13b*. IgA is the main isotype in the intestine and is responsible for maintaining intestinal homeostasis [83]. According to Inamine and Schnabl [84], any disruption in the intestine directly affects the liver because the liver receives the products absorbed in the intestine via the portal circulation. In contrast, liver tissue can alter intestinal homeostasis through the secretion of hepatic IgA and bile acids [85]. In the current study, the gene with the greatest reduction in response to PPAH supplementation was *Tgfβ1*. In mammals, *Tgfβ1* plays an important role in systemic IgA expression and production, but overproduction of *Tgfβ1* can induce fibrosis in the liver, kidney, and lung [86]. Leite et al. [87] mention that low expression of the *Cd40lg* gene decreases serum IgA levels and causes defects in T-cell proliferation. Likewise, lower expression of the *Il6* gene negatively affects IgA secretion [88]. In addition, the genes *Tnfsf13*, *H2-Oa*, *Cxcl12*, and *Tnfrsf13b* are related to B cell activating factors as IgA precursors [89].

On the other hand, dietary supplementation with PPAH decreased the expression of several genes (*Tgfβr2*, *Ltbp1*, *Tgfβ1*, *E2f5*, *Bmpr1a*, *Smurf2*, *Tgfβ2*, *Acvr2a*, *Acvr1b*, and *Inhba*) within the biological process of TGF-β signaling. Among these genes, the most biologically important are *Tgfβr1*, *Tgfβr2*, *Acvr2a*, and *Acvr1b* since, according to Utoh et al. [90], the genes of the activin family, such as *Acvr2a* and *Acvr1b*, are potent inhibitors of hepatocyte proliferation. Likewise, Morikawa et al. [86] mention that the genes *Tgfβr1* and *Tgfβr2* act as potent immunosuppressive cytokines.

Within the biological process of amoebiasis, dietary supplementation with PPAH decreased the expression of several genes, including *Il6*, *Serpinb9c*, *Rab5a*, *Muc2*, *Rab7*, *Lamb1*, and *Lama1*. Portunato et al. [91] mention that amoebiasis is an infection caused by some protozoa that reach the liver through the hepatic portal circulation and form abscesses. A low concentration of *Il6* facilitates the development of abscesses in the liver [92], while a low expression of *Serpinb9c* increases the sensitivity of hepatocytes to death by natural killer cells [93]. Verma et al. [94] state that the *Rab5a* and *Rab7* genes are required for the biogenesis of giant endocytic vacuoles in the liver. Likewise, the *Muc2* gene encodes for the mucin 2 protein, which is necessary for the synthesis of a biofilm essential for the colonization and invasion of bacteria [95]. In addition, the *Lamb1* and *Lama1* genes are related to the progression of liver fibrosis [94].

Within the biological process of non-alcoholic fatty liver disease, dietary supplementation with PPAH increased the expression of the *Lep* and *Xbp1* genes. The *Lep* gene encodes for the production of leptin, which is an anti-steatotic hormone that stimulates lipid mobilization and prevents its accumulation in the liver [96]. Likewise, the *Xbp1* gene encodes for the X-box binding protein 1, which has a hepatoprotective function against non-alcoholic steatohepatitis [97]. These effects suggest that dietary supplementation with PPAH could improve liver health in finishing lambs. The PPA used in the current study contains PtdCho, which is essential for the export of triglycerides from the liver [25]. However, non-alcoholic fatty liver disease develops when the amount of fatty acids, lipogenesis, or the synthesis of triglycerides from diglycerides exceeds the export or oxidation of fatty acids [98].

In small ruminants, when the oxidative activity of ROS exceeds the neutralizing capacity of the body’s antioxidant defense system, OS is produced [99]. According to Carvajal [100], most ROS are generated at the mitochondrial level in complexes I and III of the electron transport chain through the transfer of electrons to molecular oxygen. In the current study, within the biological processes of oxidative phosphorylation and chemical carcinogenesis, dietary supplementation with PPAH increased the expression of genes involved in the electron transport chain at the level of complex I–NADH:ubiquinone oxidoreductase (*Ndufc1*, *Ndufa1*, *Ndufb4*, *Ndufv2*, *Ndufa3*), complex III–cytochrome c reductase (*Uqcr10*, *Uqcrfs1*, *Uqcrq*), and complex IV–cytochrome c oxidase (*Cox7a2l*, *Cox6b1*). An increased expression of genes in complex I (NADH:ubiquinone oxidoreductase) of the electron chain suggests that dietary supplementation with PPAH could increase oxidative efficiency in finishing lambs since, according to Weiss et al. [101], NADH:ubiquinone oxidoreductase is one of the two rate-limiting enzymes for substrate de-electronation. Likewise, increased expression of genes in the NADH:ubiquinone oxidoreductase complex could decrease the production of some ROS (e.g., superoxides) in lamb cells since, according to Bazil et al. [102], the NADH:ubiquinone oxidoreductase complex is one of the sites with the highest electron leak before oxygen is reduced to water by cytochrome c oxidase.

On the other hand, a higher expression of the genes of the enzymatic complexes I, III, and IV of the electron transport chain is positively correlated with a higher mitochondrial respiratory capacity [103]. Ruminants’ high mitochondrial respiration rate is associated with better feed efficiency [104]. Furthermore, dietary supplementation with PPAH increased the expression of the *Atp5l* gene within oxidative phosphorylation. This effect suggests that dietary supplementation with PPAH improves metabolic energy production in lambs because the *Atp5l* gene encodes for the G subunit of mitochondrial ATP synthase, which helps to synthesize ATP from ADP and inorganic phosphate [105].

## 5. Conclusions

Dietary supplementation with 7.5 g/kg DM of PPA (BioCholine^®^) improves dry matter intake, weight gain, feed efficiency, dietary energy utilization efficiency, and *Longissimus dorsi* muscle area without altering other carcass traits, meat quality, or blood metabolites. Likewise, gene expression analysis indicates that supplementation with high doses (7.5 g/kg DM) of BioCholine^®^ could improve metabolic energy production and antioxidant status in finishing lambs.

## Figures and Tables

**Table 1:** Gas chromatographic–mass spectral analysis of the bioactive components of BioCholine^®^.

Component	Retention Time (min)	Molecular Formula	Molecular Weight (g/mol)	Proportion of Total Area (%)
3-Hexanol	3.86	C_6_H_14_O	102.1	0.15
Pentanoic acid	5.89	C_5_H_10_O_2_	102.1	0.04
Hexanoic acid	7.82	C_6_H_12_O_2_	116	0.67
Undecane	9.62	C_11_H_24_	156.1	0.06
Octanoic acid	10.86	C_8_H_16_O_2_	144.1	0.09
5-Hydroxymethylfurfural	11.65	C_6_H_6_O_3_	126	0.07
Thymoquinone	11.96	C_10_H_12_O_2_	164.1	0.16
Nonanoic acid	12.23	C^9^H^18^O_2_	158.1	0.09
Carvacrol	12.57	C_10_H_14_O_2_	150.1	1.75
n-Decanoic-acid	13.53	C_10_H_20_O_2_	172.1	0.06
p-Cymene	15.98	C_10_H_14_O_2_	166.1	0.13
Dodecanoic acid	16.05	C_12_H_24_O_2_	200.2	0.46
Tetradecanoic acid	18.31	C_14_H_28_O_2_	228.2	1.31
Pentadecanoic acid	19.31	C_15_H_30_O_2_	242.2	0.33
Hexadecanoic acid, methyl ester	19.98	C_17_H_34_O_2_	270.3	0.65
n-Hexadecanoic acid, palmitico	20.48	C_16_H_32_O_2_	256.2	16.24
12-Octadecadienoic acid, methyl ester	21.62	C_19_H_34_O_2_	1.77	1.77
9-Octadecenoic acid, methyl ester	21.68	C_19_H_36_O_2_	1.44	1.44
Oleic acid	21.90	C_18_H_34_O_2_	282.3	0.98
12-Octadecanoic acid, linoleico	22.33	C_18_H_32_O_2_	280.2	58.94

**Table 2:** Growth performance of finishing lambs supplemented with a polyherbal phytogenic additive ^1^.

Parameter	Treatments				SEM			*p*-Value	
	CON	PPAL	PPAM	PPAH		Per	Trat × Per	Linear	Quadratic
Initial body weight, kg	23.22	23.91	23.52	23.78	0.597	-	-	-	-
Final body weight, kg	40.49	41.35	41.96	44.32	1.573	<0.0001	0.99	0.08	0.73
Daily weight gain (DWG), kg/d	0.308 ^b^	0.311 ^b^	0.329 ^b^	0.367 ^a^	0.015	<0.0001	0.88	0.01	0.33
Dry matter intake (DMI), kg/d	1.333 ^b^	1.328 ^b^	1.344 ^ab^	1.407 ^a^	0.032	<0.0001	0.95	0.02	0.27
DMI variation (%) ^1^	12.24	14.01	14.10	12.09	1.268	<0.0001	0.29	0.93	0.11
Feed conversion ratio (FCR), DMI/DWG	5.06 ^a^	4.85 ^ab^	4.37 ^bc^	4.22 ^c^	0.218	<0.0001	0.33	0.02	0.92
Observed dietary net energy, Mcal/kg									
Maintenance (ObsNEm)	1.793 ^b^	1.840 ^b^	1.892 ^ab^	2.003 ^a^	0.080	<0.0001	0.85	0.02	0.63
Gain (ObsNEg)	1.162 ^b^	1.204 ^b^	1.250 ^ab^	1.346 ^a^	0.070	<0.0001	0.85	0.02	0.63
Observed to expected diet net energy									
Maintenance (OExNEm)	0.991 ^b^	1.017 ^b^	1.046 ^ab^	1.106 ^a^	0.044	<0.0001	0.85	0.02	0.63
Gain (OExNEg)	0.922 ^b^	0.955 ^b^	0.992 ^ab^	1.068 ^a^	0.039	<0.0001	0.85	0.02	0.63
Observed to expected DMI	1.128 ^a^	1.110 ^a^	1.037 ^ab^	0.976 ^b^	0.059	<0.0001	0.75	0.01	0.65

BioCholine^®^ based on *Trachyspermum ammi*, *Andrographis paniculata*, *Achyrantes aspera*, and *Azadirachta indica*. ^1^ Variation in daily feed intake among individuals between days; CON—basal diet without polyherbal phytogenic additive (PPA); PPAL—basal diet + 2.5 g of PP/kg DM; PPAM—basal diet + 5 g of PPA/kg DM; PPAH—basal diet + 7.5 g of PPA/kg DM. SEM—standard error of the treatment means; ^a,b,c^—means within a row with different subscripts differ when *p* ≤ 0.05.

**Table 3:** Carcass traits of finishing lambs supplemented with a polyherbal phytogenic additive ^1^.

Parameter	Treatments				SEM	*p*-Value	
	CON	PPAL	PPAM	PPAH		Linear	Quadratic
Back fat thickness (BFT), mm	4.20	4.24	4.33	4.37	0.08	0.10	0.98
Muscle area *longissimus dorsi* (LDMA), cm^2^	10.11 ^c^	10.50 ^bc^	10.73 ^b^	11.23 ^a^	0.16	<0.001	0.70
Hot carcass weight (HCW), kg	20.25	20.90	20.62	20.86	0.72	0.17	0.68
Hot carcass yield (HCY), %	50.26	50.74	49.48	29.29	0.99	0.35	0.74
Cold carcass weight (CCW), kg	18.62	19.31	19.26	20.37	0.77	0.14	0.79
Cold carcass yield (CCY), %	46.20	46.63	46.24	45.91	0.98	0.78	0.70
Yield grade (YG)	0.41	0.41	0.42	0.42	0.03	0.10	0.98
External length of the carcass (ELC), cm	57.33	57.44	57.33	58.33	0.78	0.41	0.57
Internal length of the carcass (ILC), cm	53.89	55.22	55.00	54.44	0.72	0.65	0.20
Chest girth (CG), cm	71.78	74.44	73.44	75.11	1.21	0.67	0.34
Length of the leg (LL), cm	32.22	33.77	33.00	33.33	0.59	0.83	0.78
Perimeter of the leg (PL), cm	34.66	35.00	36.88	36.89	0.63	0.10	0.79
Compactness index (CI), kg/cm	0.34	0.35	0.35	0.37	0.01	0.10	0.41

^1^ BioCholine^®^ based on *Trachyspermum ammi*, *Andrographis paniculata*, *Achyrantes aspera*, and *Azadirachta indica*. CON—basal diet without polyherbal phytogenic additive (PPA); PPAL—basal diet + 2.5 g of PP/kg DM; PPAM—basal diet + 5 g of PPA/kg DM; PPAH—basal diet + 7.5 g of PPA/kg DM. SEM—standard error of the treatment means; ^a,b,c^—means within a row with different subscripts differ when *p* ≤ 0.05.

**Table 4:** Organ weights of finishing lambs supplemented with a polyherbal phytogenic additive ^1^.

Parameter	Treatments				SEM	*p*-Value	
	CON	PPAL	PPAM	PPAH		Linear	Quadratic
Stomach complex (empty), kg	1.451	1.406	1.441	1.512	0.068	0.48	0.40
Small intestine (empty), kg	0.887	0.896	0.926	0.965	0.054	0.08	0.28
Large intestine (empty), kg	1.040	1.117	1.291	1.222	0.136	0.24	0.11
Lungs and trachea, kg	0.639	0.666	0.635	0.646	0.042	0.96	0.85
Heart, kg	0.191	0.190	0.179	0.200	0.013	0.81	0.39
Liver, kg	0.898	0.897	0.899	0.882	0.049	0.84	0.87
Kidneys, kg	0.634	0.669	0.683	0.714	0.048	0.46	0.33
Spleen, kg	0.079	0.084	0.078	0.076	0.006	0.59	0.57
Testicles, kg	0.565	0.592	0.594	0.586	0.046	0.12	0.15
Skin, kg	3.305	3.197	3.077	3.436	0.157	0.70	0.14
Head, kg	2.253	2.158	2.124	2.269	0.087	0.97	0.18
Feet, kg	0.965	0.889	0.913	0.987	0.045	0.96	0.11

^1^ BioCholine^®^ based on *Trachyspermum ammi*, *Andrographis paniculata*, *Achyrantes aspera*, and *Azadirachta indica*. CON—basal diet without polyherbal phytogenic additive (PPA); PPAL—basal diet + 2.5 g of PP/kg DM; PPAM—basal diet + 5 g of PPA/kg DM; PPAH—basal diet + 7.5 g of PPA/kg DM. SEM—standard error of the treatment means.

**Table 5:** Meat traits of finishing lambs supplemented with a polyherbal phytogenic additive ^1^.

Parameter	Treatments				SEM	*p*-Value	
	CON	PPAL	PPAM	PPAH		Linear	Quadratic
Cooking loss (CL), %	17.47	16.50	16.65	18.73	1.45	0.29	0.17
Lightness (L*)	34.95	34.02	34.57	35.69	1.23	0.96	0.26
Redness (a*)	10.47	10.45	9.40	10.60	0.50	0.76	0.22
Yellowness (b*)	10.08	9.93	10.22	10.94	0.68	0.73	0.12
Protein, g/100 g	19.73	19.80	20.19	19.98	0.17	0.22	0.17
Fat, g/100 g	3.29	3.44	3.19	3.03	0.27	0.77	0.10
Moisture, g/100 g	75.05	74.68	74.62	74.85	0.33	0.41	0.16
Collagen, g/100 g	1.71	1.64	1.62	1.71	0.06	0.88	0.11

^1^ BioCholine^®^ based on *Trachyspermum ammi*, *Andrographis paniculata*, *Achyrantes aspera*, and *Azadirachta indica*. CON—basal diet without polyherbal phytogenic additive (PPA); PPAL—basal diet + 2.5 g of PP/kg DM; PPAM—basal diet + 5 g of PPA/kg DM; PPAH—basal diet + 7.5 g of PPA/kg DM. SEM—standard error of the treatment means.

**Table 6:** Hematological profile of finishing lambs supplemented with a polyherbal phytogenic additive ^1^.

Parameter	Treatments				SEM	*p*-Value	
	CON	PPAL	PPAM	PPAH		Linear	Quadratic
Hematocrit, %	35.44	38.00	36.11	36.44	1.30	0.85	0.39
Hemoglobin, g/dL	11.79	12.05	11.76	12.20	0.26	0.42	0.73
Red blood cells, 10^6^/mL	8.27	8.38	8.63	9.15	0.34	0.08	0.36
Mean corpuscular volume, Fl	29.27	29.49	20.40	29.46	0.51	0.84	0.87
Mean corpuscular hemoglobin, pg	14.07	15.10	13.78	13.32	0.80	0.33	0.36
Mean corpuscular hemoglobin concentration, g/dL	48.31	51.66	47.10	45.57	2.86	0.32	0.39
Platelets, 10^3^/mL	936.00	986.11	826.33	1004.67	52.22	0.84	0.22
Leukocytes, 10^3^/mL	9.01	9.19	8.71	8.25	0.43	0.15	0.45
Lymphocytes, 10^3^/mL	45.66	46.00	47.22	48.55	3.48	0.80	0.88
Monocytes, 10^3^/mL	8.19	10.67	9.33	8.89	1.36	0.29	0.07
Segmented neutrophils, 10^3^/mL	43.67	42.33	43.00	45.78	3.76	0.67	0.58
Band neutrophils, 10^3^/mL	0	0	0	0	0	0	0
Eosinophils, 10^3^/mL	2.67	1.00	0.89	1.33	0.62	0.14	0.09
Basophils, 10^3^/mL	0.11	0.00	0.00	0.00	0.05	0.18	0.32
Plasma protein, g/dL	6.79	6.70	6.85	6.67	0.17	0.82	0.79

^1^ BioCholine^®^ based on *Trachyspermum ammi*, *Andrographis paniculata*, *Achyrantes aspera*, and *Azadirachta indica*. CON—basal diet without polyherbal phytogenic additive (PPA); PPAL—basal diet + 2.5 g of PP/kg DM; PPAM—basal diet + 5 g of PPA/kg DM; PPAH—basal diet + 7.5 g of PPA/kg DM. SEM—standard error of the treatment means.

**Table 7:** Biochemistry of blood serum of finishing lambs supplemented with a polyherbal phytogenic additive ^1^.

Parameter	Treatments				SEM	*p*-Value	
	CON	PPAL	PPAM	PPAH		Linear	Quadratic
Glucose, mg/dL	65.44 ^a^	64.12 ^ab^	60.00 ^b^	58.35 ^b^	1.93	0.05	0.38
Cholesterol, mg/dL	40.33	37.11	45.77	37.11	4.06	0.95	0.50
Triglycerides, mg/dL	18.77	17.88	19.89	17.33	1.81	0.77	0.55
Urea, mg/dL	42.11	44.44	47.89	40.33	3.17	0.89	0.12
Uric acid, mg/dL	0.47 ^a^	0.46 ^a^	0.25 ^b^	0.32 ^b^	0.04	0.001	0.35
Creatinine, mg/dL	0.87 ^a^	0.78 ^a^	0.62 ^b^	0.46 ^c^	0.05	<0.0001	0.55
Total protein, g/dL	5.45	5.23	5.48	5.19	0.35	0.74	0.91
Albumin, g/dL	2.82	2.74	2.88	2.84	0.16	0.78	0.90
Globulin, g/dL	2.62	2.48	2.60	2.46	0.18	0.63	0.99
Albumin/globulin	1.08	1.10	1.11	1.13	0.03	0.36	0.92
Bilirubin, mg/dL	0.51 ^a^	0.50 ^a^	0.39 ^b^	0.28 ^b^	0.05	0.006	0.43
Alkaline phosphatase, UI/dL	463.55	451.00	481.00	486.11	48.91	0.65	0.85
Lactate dehydrogenase, UI/dL	395.78	434.67	445.89	394.78	29.59	0.89	0.11
Aspartate aminotransferase, UI/dL	87.78	95.55	93.55	90.67	6.89	0.83	0.44
Calcium, mg/dL	9.24	8.63	9.44	9.35	0.32	0.43	0.42
Phosphorus, mg/dL	3.84	3.69	3.87	3.99	0.15	0.36	0.38

^1^ BioCholine^®^ based on *Trachyspermum ammi*, *Andrographis paniculata*, *Achyrantes aspera*, and *Azadirachta indica*. CON—basal diet without polyherbal phytogenic additive (PPA); PPAL—basal diet + 2.5 g of PP/kg DM; PPAM—basal diet + 5 g of PPA/kg DM; PPAH—basal diet + 7.5 g of PPA/kg DM. SEM—standard error of the treatment means; ^a,b,c^—means within a row with different subscripts differ when *p* ≤ 0.05.

**Table 8:** Biological processes enriched with differentially down-expressed genes in liver tissue from lambs supplemented with PPAH.

**DNA Replication (FC = 4.7; *p*-Value = 0.003)**			**Tyrosine Metabolism (FC = 4.1; *p*-Value = 0.006)**		
**Symbol**	**Gene Name**	**FC**	**Symbol**	**Gene Name**	**FC**
Prim2	DNA primase, p58 subunit	−2.81	Aldh3a1	Aldehyde dehydrogenase family 3, subfamily A1	−2.62
Rfc1	Replication factor C (activator 1) 1	−2.31	Adh4	Alcohol dehydrogenase 4 (class II), pi polypeptide	−2.15
Mcm4	Minichromosome maintenance complex component 4	−2.01	Aox1	Aldehyde oxidase 1	−2.14
Rnaseh2b	Ribonuclease H2, subunit B	−1.94	Fahd1	Fumarylacetoacetate hydrolase domain containing 1	−2.09
Pold1	Polymerase (DNA directed), delta 1, catalytic subunit	−1.83	Got2	Glutamatic-oxaloacetic transaminase 2, mitochondrial	−1.86
Rfc4	Replication factor C (activator 1) 4	−1.55	Maoa	Monoamine oxidase A	−1.77
Rfc3	Replication factor C (activator 1) 3	−1.54	4930438A08Rik	RIKEN cDNA 4930438A08 gene	−1.60
**Drug metabolism—Cytochromo P450 (FC = 3.3; *p*-Value = 0.003)**			**TGF-β Signaling Pathway (FC = 2.7; *p*-Value = 0.007)**		
**Symbol**	**Gene Name**	**FC**	**Symbol**	**Gene Name**	**FC**
Gsta3	Glutathione S-transferase, alpha 3	−2.86	Acvr2a	Activin receptor IIA	−2.78
Aldh3a1	Aldehyde dehydrogenase family 3, subfamily A1	−2.62	Tgfbr2	Transforming growth factor, beta receptor II	
Gsta4	Glutathione S-transferase, alpha 4	−2.56	Ltbp1	Latent transforming growth factor beta binding protein 1	−2.50
Gstm3	Glutathione S-transferase, mu 3	−2.50	Tgfb1	Transforming growth factor, beta 1	−3.31
Adh4	Alcohol dehydrogenase 4 (class II), pi polypeptide	−2.15	E2f5	E2F transcription factor 5	−1.93
Aox1	Aldehyde oxidase 1	−2.14	Inhba	Inhibin beta-A	−1.80
Fmo1	Flavin containing monooxygenase 1	−1.91	Acvr1b	Activin A receptor, type 1B	−1.79
Maoa	Monoamine oxidase A	−1.77	Rbl1	RB transcriptional corepressor like 1	−1.76
Fmo2	Flavin containing monooxygenase 2	−1.60	Bmpr1a	Bone morphogenetic protein receptor, type 1A	−1.69
			Tgfb2	Transforming growth factor, beta 2	−1.64
**Intestinal Immune Network for Immunoglobulin A Production (FC = 3.8; *p*-Value = 0.009)**			**Amoebiasis (FC = 2.6; *p*-Value = 0.006)**		
**Symbol**	**Gene Name**	**FC**	**Symbol**	**Gene Name**	**FC**
Tgfb1	Transforming growth factor, beta 1	−3.31	Il6	Interleukin 6	−2.30
Cd40lg	CD40 ligand	−2.84	Serpinb9c	Serine (or cysteine) peptidase inhibitor, clade B, member 9c	−2.28
Il6	Interleukin 6	−2.30	Rab5a	RAB5A, member RAS oncogene family	−2.21
Tnfsf13	Tumor necrosis factor (ligand) superfamily, member 13	−1.93	Tgfb1	Transforming growth factor, beta 1	−3.31
H2-Oa	Histocompatibility 2, O region alpha locus	−1.77	Gnaq	Guanine nucleotide-binding protein, alpha q polypeptide	−1.75
Cxcl12	Chemokine (C-X-C motif) ligand 12	−1.62	Muc2	Mucin 2	−1.99
Tnfrsf13b	Tumor necrosis factor receptor superfamily, member 13b	−1.55	Rab7	RAB7, member RAS oncogene family	−1.84
			Lamb1	Laminin B1	−1.69
			Gna11	Guanine nucleotide-binding protein, alpha 11	−1.67
			Tgfb2	Transforming growth factor, beta 2	−1.64
			Lama1	Laminin, alpha 1	

PPAH—basal diet + 7.5 g of polyherbal phytogenic additive (PPA)/kg DM; PPA—based on *Trachyspermum ammi*, *Andrographis paniculata*, *Achyrantes aspera*, and *Azadirachta indica*; FC—fold change.

**Table 9:** Biological processes enriched with differentially upregulated genes in liver tissue from lambs supplemented with PPAH.

**Non-Alcoholic Fatty Liver Disease (FC = 3.5; *p*-Value = 0.00003)**			**Oxidative Phosphorylation (FC = 2.8; *p*-Value = 0.003)**		
**Symbol**	**Gene Name**	**FC**	**Symbol**	**Gene Name**	**FC**
Ndufc1	NADH:ubiquinone oxidoreductase subunit C1	2.66	Ndufc1	NADH:ubiquinone oxidoreductase subunit C1	2.66
Ndufa1	NADH:ubiquinone oxidoreductase subunit A1	2.47	Ndufa1	NADH:ubiquinone oxidoreductase subunit A1	2.47
Jun	Jun proto-oncogene	2.25	Cyct	Cytochrome c, testis	2.22
Cyct	Cytochrome c, testis	2.22	Uqcr10	Ubiquinol-cytochrome c reductase, complex III subunit X	1.99
Rxra	Retinoid X receptor alpha	2.06	Ndufb4	NADH:ubiquinone oxidoreductase subunit B4	1.90
Uqcr10	Ubiquinol-cytochrome c reductase, complex III subunit X	1.99	Uqcrfs1	Ubiquinol-cytochrome c reductase, Rieske iron-sulfur polypeptide 1	1.87
Ndufb4	NADH:ubiquinone oxidoreductase subunit B4	1.90	Ndufv2	NADH:ubiquinone oxidoreductase core subunit V2	1.85
Uqcrfs1	Ubiquinol-cytochrome c reductase, Rieske iron-sulfur polypeptide 1	1.87	Uqcrq	Ubiquinol-cytochrome c reductase, complex III subunit VII	1.78
Ndufv2	NADH:ubiquinone oxidoreductase core subunit V2	1.85	Ndufa3	NADH:ubiquinone oxidoreductase subunit A3	1.77
Pik3cd	Phosphatidylinositol-4,5-bisphosphate 3-kinase catalytic subunit delta	1.83	Atp5l	ATP synthase, H+ transporting, mitochondrial F0 complex, subunit G	1.70
Xbp1	X-box binding protein 1	1.79	Cox7a2l	Cytochrome c oxidase subunit 7A2-like	1.55
Uqcrq	Ubiquinol-cytochrome c reductase, complex III subunit VII	1.78	Cox6b1	Cytochrome c oxidase, subunit 6B1	1.51
Ndufa3	NADH:ubiquinone oxidoreductase subunit A3	1.77			
Lep	Leptin	1.59			
Cox7a2l	Cytochrome c oxidase subunit 7A2-like	1.55			
Cox6b1	Cytochrome c oxidase, subunit 6B1	1.51			
**Chemical Carcinogenesis–Reactive Oxygen Species (FC = 2.4; *p*-Value = 0.002)**					
**Symbol**	**Gene Name**				**FC**
Ndufc1	NADH:ubiquinone oxidoreductase subunit C1				2.66
Sos2	SOS Ras/Rho guanine nucleotide exchange factor 2				2.50
Ndufa1	NADH:ubiquinone oxidoreductase subunit A1				2.47
Raf1	V-raf-leukemia viral oncogene 1				2.46
Mapk9	Mitogen-activated protein kinase 9				2.42
Jun	Jun proto-oncogene				2.25
Uqcr10	Ubiquinol-cytochrome c reductase, complex III subunit X				1.99
Gstm2	Glutathione S-transferase, mu 2				1.94
Ndufb4	NADH:ubiquinone oxidoreductase subunit B4				1.90
Uqcrfs1	Ubiquinol-cytochrome c reductase, Rieske iron-sulfur polypeptide 1				1.87
Ndufv2	NADH:ubiquinone oxidoreductase core subunit V2				1.85
Pik3cd	Phosphatidylinositol-4,5-bisphosphate 3-kinase catalytic subunit delta				1.83
Uqcrq	Ubiquinol-cytochrome c reductase, complex III subunit VII				1.78
Ndufa3	NADH:ubiquinone oxidoreductase subunit A3				1.77
Ptpn11	Protein tyrosine phosphatase, non-receptor type 11				1.58
Cox7a2l	Cytochrome c oxidase subunit 7A2-like				1.55
Cox6b1	Cytochrome c oxidase, subunit 6B1				1.51

PPAH—basal diet + 7.5 g of polyherbal phytogenic additive (PPA)/kg DM; PPA—based on *Trachyspermum ammi*, *Andrographis paniculata*, *Achyrantes aspera*, and *Azadirachta indica*; FC—fold change.

## Data Availability

The datasets used and analyzed during the current study are available from the corresponding author upon reasonable request.

## Appendix A

**Table A1:** Ingredients and nutrient composition of the basal diets.

Ingredients, g/kg of DM	Treatments			
	CON	PPAL	PPAM	PPAH
Oat straw	194	194	194	194
Ground corn	303	300.5	298	295.5
Ground sorghum	241	241	241	241
Soybean meal	81	81	81	81
Wheat bran	71	71	71	71
Corn gluten	74	74	74	74
Bypass fat	23	23	23	23
Common salt	5	5	5	5
Vitamin and mineral premix ^a^	5	5	5	5
Calcium carbonate	3	3	3	3
Polyherbal phytogenic additive (PPA) ^b^	0	2.5	5	7.5
Total	1000	1000	1000	1000
**Nutrient composition, g/kg of DM**				
Dry matter	892.3	893.2	888.0	889.5
Crude protein	158.1	157.9	157.7	157.4
Ether extract	25.5	25.4	25.3	25.3
Neutral detergen fiber	271.2	273.8	272.3	276.2
Acid detergent fiber	142.1	143.4	142.8	144.6
Ashes	55.2	54.2	49.6	50.1
Calculated net energy, Mcal/kg				
Maintenance ^c^	1.79	1.79	1.79	1.79
Gain ^c^	1.25	1.25	1.25	1.25

CON: basal diet without polyherbal phytogenic additive (PPA); PPAL: basal diet + 2.5 g of PPA/kg of DM; PPAM: basal diet + 5 g of PPA/kg of DM; PPAH: basal diet + 7.5 g of PPA/kg of DM; ^a^ vitamin and mineral premix (Ovino plus, Vitasal^®^, Texcoco, Mexico): calcium, 24%; chlorine, 12%; sodium, 8%; phosphorus, 3%; magnesium, 2%; potassium, 0.5%; sulfur, 0.5%; antioxidant, 0.5%; Lasalocid, 2000 ppm; zinc, 5000 ppm; manganese, 4000 ppm; iron, 2000 ppm; iodine, 100 ppm; selenium, 30 ppm; cobalt, 60 ppm; chromium, 5 ppm; vitamin A, 500 000 UI; vitamin D, 150 000 UI; vitamin E, 1000 UI. ^b^ BioCholine^®^ based on *Trachyspermum ammi*, *Andrographis paniculata*, *Achyrantes aspera, and Azadirachta indica*. ^c^ The calculation is based on NRC [28].
